# Allergic Rhinitis and House Dust Mite Sensitization Determine Persistence of Asthma in Children

**DOI:** 10.1007/s12098-021-04052-5

**Published:** 2022-03-04

**Authors:** A. Brzozowska, K. Woicka-Kolejwa, J. Jerzynska, P. Majak, I. Stelmach

**Affiliations:** 1 Department of Pediatrics and Allergy, N. Copernicus Hospital, Medical University of Lodz, 71 Al. Pilsudskiego, 90-329 Lodz, Poland; 2 Department of Internal Medicine, Asthma and Allergy, N. Copernicus Hospital, Medical University of Lodz, Lodz, Poland

**Keywords:** Children, Asthma persistence, Allergic rhinitis, House dust mite sensitization

## Abstract

**Objective:**

To specify clinical and immunological parameters of the mechanisms, which may lead to development of persistent asthma, or regression of the disease symptoms.

**Methods:**

Eighty children with childhood asthma, diagnosed in the past by using the modified Asthma Predicted Index (mAPI), were divided into two groups: remission group and persistent group. There were 3 study visits (baseline, at 6 mo, and at 12 mo). Clinical remission of asthma was defined as the absence of asthma symptoms for at least 12 mo without treatment. The patients could switch from one group to another during the 12 mo of follow-up. Clinical, inflammatory, and immunoregulatory predictors of asthma remission/persistence were analyzed.

**Results:**

The presence of mAPI criteria as well as house dust mite (HDM) allergy and allergic rhinitis at 7–10 y, were associated with a reduced prevalence of asthma remission. The increased eosinophil blood count in mAPI criteria was associated with a lower expression of CD25 positive cells. HDM allergy was associated with a higher fractional exhaled nitric oxide (FeNO) level (*p* = 0.0061) and higher expression of CD25CD71 (*p* = 0.0232). Allergic rhinitis was associated with a higher expression of PPAR (*p* = 0.0493) and CD25CD71 (*p* = 0.0198), and lower expression of glycoprotein A repetitions predominant (GARP).

**Conclusions:**

Persistence of childhood asthma was largely determined by the presence of allergic rhinitis and sensitization to HDM. Additionally, API criteria but not immunoregulation processes, were related to asthma persistence.

**Supplementary Information:**

The online version contains supplementary material available at 10.1007/s12098-021-04052-5.

## Introduction

Symptoms of asthma in adults may have started in childhood [1]. It was revealed by the Multicenter Allergy study, that the chronic asthma is characterized by airway hyper-responsiveness and impairment of lung function at school age. What is more, it is determined by continuing allergic airway inflammation, which begins in the first 3 y of life. Moreover, symptom relief has been observed in children with a nonatopic wheezing phenotype over school age and restoration of the normal lung function at adolescence [2]. Furthermore, increased risk of allergic disorders may be induced by respiratory tract infections in early childhood, and allergic comorbidities, exposure to environmental determinants of asthma and active smoking both in adolescence and adulthood [1, 3]. There is little understanding of the immunological mechanisms by which asthma develops into a persistent disease, or by which symptoms regress.

The induction and maintenance of tolerance is broadly influenced by a wide range of cell types and suppressive molecules. The expression of fork transcription factor (FOXP3) in regulatory T lymphocytes (Tregs) plays a key role in maintaining immune tolerance [4, 5]. IL-2 receptor (CD25) and transferrin receptor (CD71) are markers of T and B lymphocyte activation. Increased expression of these molecules might reflect an increased activation state [3, 6]. Moreover, activation of transcription factors, such as the suppressor of cytokine signaling (SOCS), and peroxisome proliferator-activated receptor gamma (PPARγ), also may have an influence on the course of inflammation (PPARγ exerts anti-inflammatory effects) [6, 7]. The expression of cytokine signaling 3 (SOCS3) is induced by various cytokines, including IL-6 and IL-10 [8, 9]. The glycoprotein A repetitions predominant (GARP) is involved in peripheral tolerance (TGF-β1 activation) [10]. The hypothesis in the present study was that in these processes some cells’ mediators might play more significant role than others.

This study was designed to investigate the clinical and immunological parameters that may be associated with the resolution of asthma symptoms. Children with persistent asthma and those whose childhood asthma symptoms resolved were compared on a number of criteria, including clinical data, API (predictive index of asthma) criteria, and immunoregulatory parameters. It was expected that the immunological proinflammatory parameters would decrease in patients with asthma remission, and clinical parameters, for example fractional exhaled nitric oxide (FeNO), would improve in this group of patients.

## Material and Methods

This was a prospective study based on 80 children, aged 7–10 y, enrolled between September 2018 and May 2019. Participants were diagnosed with bronchial asthma < 5 y of age and remained under the care of the authors' clinic. Patients with concomitant diseases were excluded from the study.

Parents were requested to attend the clinic with their children by phone call. At the first visit they were informed about the purpose of the study and underwent skin prick testing. Demographic data on gender, type of residence, exposure to molds, pets at home, exposure to tobacco, perinatal history (type of delivery, Apgar score, birth weight), and family and medical history were collected; patients were qualified as atopics bias, on history and positive skin tests. The measurements of serum specific IgE were applied whenever a patient was taking antihistamines or skin testing was impossible due to the lack of cooperation. At this visit, children were divided into two groups: remission and persistent group. The first group included children in whom asthma treatment was not continued by the allergist from the authors' outpatient clinic due to the absence of clinical symptoms of the disease. It was confirmed by the statement of the family, lung function tests, and exhaled nitric oxide (NO) concentration. No ICS, SABA, nor LTRAs were allowed (patients with allergic rhinitis were allowed to take nasal steroids). The second group was characterized by the children who remained on antiasthmatic therapy due to asthma symptoms. To form remission and persistent groups with 40 children in each one (80 children in total), 100 children were screened. The patients could switch from group of remission to the persistent group during the 12 mo of follow-up. The second visit took place 6 mo after the first one, and another third visit took place 12 mo from the first visit. At each visit, to confirm asthma status in the patients, lung function and exhaled NO concentration were measured. At the third visit, blood samples to assess immunological parameters were obtained from all patients.

The early childhood asthma (< 5 y of age) was diagnosed by at least 1-y observation by the clinician. It was based on asthma symptoms (wheezing, recurrent lower respiratory tract infections, dyspnea) and using the modified Asthma Predicted Index (mAPI). Criteria used by mAPI require 4 or more wheezing episodes in the last year, in addition to one of the three major criteria: physician-diagnosed parental asthma, physician-diagnosed child eczema, and sensitization to ≥ 1 aeroallergen, or two out of the three minor criteria: wheezing apart from colds, blood eosinophils ≥ 4%, and sensitization to ≥ 1 food allergen [11, 12]. Children were followed up and taken care of by the authors' allergy outpatient clinic.

Clinical remission of asthma in the patient under study was defined as the absence of asthma symptoms for at least 12 mo. What is more, during this time the patient could not use inhaled corticosteroids and short acting beta 2 agonists. The spirometry and reversibility test of the patient confirmed complete remission as previously described [12–14].

Skin prick testing to standard allergen extracts was performed by using Allergopharma, Reinbek, Germany. “If a reaction > 3 mm in diameter has formed within 15 min above the negative control, it was considered positive. Allergen sensitization was defined as specific IgE of ≥ 0.35 KU/L for at least one of the tested allergens (chemiluminescence method (CLIA), Immulite 2000, XPI, Siemens, Germany)” [15].

“All pulmonary function tests were performed with a Master Screen unit (Erich Jaeger Gmbh-Hochberg, Germany), as described elsewhere, in accordance with the ATS/ERS guidelines” [15, 16].

“Fractional exhaled nitric oxide (FeNO) was measured with a chemiluminescence analyzer (Sievers NOA 280i, CO, USA). Exhalations were performed in accordance with the ATS/ERS guidelines” [17].

The following panel of antibodies conjugated with fluorescein isothiocyanate (FITC), phycoerythrin (PE), peridinin-chlorophyll-protein (PerCP), or allophycocyanin (APC), was used for the following assays: PPARG-FITC, CD11c-PE, CD 25 FITC, CD 4 PerCP, CD 71 PE, CD 73, PerCP, anti-GARP APC, FOXP3 PE, (Becton Dickinson, San Diego, CA, USA), and SOCS3 (Abbexa, Cambridge, UK). All procedures were carried out according to the manufacturer's instructions; the detailed description is included in Supplementary material S1.

The study was approved by the Medical Ethics Committee. All parents/legal guardians gave their oral and written consent for the evaluation of data from the medical documentation of their children.

Statistical analysis was conducted in three steps. The first and second step comprised the logistic regression analysis in univariate followed by the multivariate model. Significant (*p* < 0.1) predictors of asthma remission defined in univariate models were included into the final multivariate model. During the third step, immunological parameters were compared in relation to the presence or absence of previously defined clinical predictors of asthma remission. The significance threshold of *p* level was set at 0.05. The statistical analysis was performed with STATISTICA 13.1 (StatSoft Poland, Kraków).

## Results

Eighty patients were included in the analysis. Three children in the remission group at 6 mo ended up being in the persistent group during the 12-mo visit and they were excluded from the study. Therefore, 3 new patients were enrolled in the study to fill up the gap. Clinical characteristics of study groups are presented in Table 1.

**Table 1:** Baseline characteristics

	Total group *N* = 80		Asthma persistence *N* = 40		Asthma remission *N* = 40		*p*
	*N*	%	*N*	%	*N*	%	
Age (years) at time of diagnosis; mean (SD)	4.1 (2.1)						
Age (years) at present; mean SD	8.2 (2.0)		8.4 (1.9)		7.9 (1.9)		0.1871
Male gender, *N* (%)	43	53.8	21	52	22	55	0.8225
BMI (kg/m^2^); mean (SD)	18.4 (3.9)		18.8 (4.5)		17.9 (3.1)		0.5819
Early life data							
Preterm delivery, *N* (%)	8	10	6	15	2	5	0.1281
Cesarean delivery, *N* (%)	31	38.75	15	37	16	40	0.8185
Apgar (points); median	10 (9 to 10)		9.5 (9 to 10)		10 (9 to 10)		0.4182
Birth weight (g); median (quartile range)	3225 (2910 to 3600)		3225 (2920 to 3600)		3240 (2880 to 3600)		0.9985
API index, N (%)							
API atopic dermatitis	53	66.3	27	67	26	65	0.8131
API sensitization to allergens	51	63.8	31	77	20	50	**0.0098**
API parental asthma	36	45.0	22	55	14	35	0.0712
API eosinophils	28	35.0	19	47	9	22	**0.0181**
API wheezing	61	76.3	35	87	26	65	**0.0163**
API food allergy	41	51.3	20	50	21	52	0.8230
Environment, N (%)*							
ETS	20	25.0	9	22	11	27	0.4710
Animals at home	48	60.0	27	67	21	52	0.1699
Molds at home	4	5.0	2	5	2	5	1.0
Allergy profile, N (%)*							
Cat	19	24.1	10	25	9	22	0.8415
Dog	4	5.1	1	2.5	3	7.5	0.2827
HDM	31	39.2	20	50	11	27	**0.0461**
Molds	10	12.7	7	17	3	7.5	0.1842
Grass	30	38.0	19	47	11	27	0.0759
Tree	18	22.8	9	22	9	22	0.9513
Food	17	21.5	10	25	7	17	0.4447
Positive family history, N (%)*	34	43.0	21	52	13	32	0.0843
Mothers’ allergy	19	24.1	11	27	8	20	0.4667
Fathers’ allergy	16	20.3	11	27	5	12	0.1009
Comorbidities, N (%)*							
Atopic dermatitis	17	21.3	10	25	7	17	0.4113
Allergic rhinitis	45	56.3	33	82	12	30	** < 0.0001**
Allergic rhinoconjuctivitis	15	18.8	14	35	1	2.5	** < 0.0001**

*API* Asthma Predictive Index; *BMI* Body mass index; *ETS* Exposure to tobacco smoke; *HDM* House dust mite allergy; *SD* Standard deviation

^*^at present

All available clinical data as independent variables were included in the univariate model of logistic regression analysis together with asthma remission as a dependent variable. Statistically significant relations in API for sensitization to allergens (OR: 0.29; 95% Cl: 0.11 to 0.76; *p* < 0.0122) were found, with very high significance for allergic rhinitis (AR) and rhinoconjuctivitis (OR: 0.09; 95% Cl: 0.03 to 0.26; *p* < 0.0001 and OR: 0.05; 95% Cl: 0.01 to 0.38; *p* < 0.0043, respectively), especially when house dust mite (HDM) allergy was confirmed (OR: 0.39; 95% Cl: 0.15 to 1.00; *p* < 0.0497). Significant correlations were also found between asthma remission and blood eosinophillia (OR: 0.32; 95% Cl: 0.12 to 0.84; *p* < 0.0213), and wheezing apart from colds (OR: 0.27; 95% Cl: 0.08 to 0.83; *p* < 0.0226) (Table 2). The presence of mAPI criteria was associated with decreased likelihood of asthma remission. In addition, the current trend toward parental asthma API and parental asthma was found to be associated with a lower likelihood of asthma remission (*p* = 0.0743 and *p* = 0.0876, respectively) (Table 2).

**Table 2:** Associations between asthma remission, defined as dependent variable and group of independent variables in univariate model of logistic regression analysis

Independent variable	OR^a^	95% CI		*p*
Age (continuous variable)	0.88	0.70	1.10	0.2589
Male gender	1.11	0.46	2.66	0.8226
API atopic dermatitis	0.89	0.35	2.26	0.8131
API sensitization to allergens	0.29	0.11	0.76	**0.0122**
API parental asthma	0.44	0.18	1.08	0.0743
API eosinophils	0.32	0.12	0.84	**0.0213**
API wheezing	0.27	0.08	0.83	**0.0226**
API food allergy	1.11	0.46	2.66	0.8230
Weight	1.00	1.00	1.00	0.8441
Apgar	1.33	0.85	2.09	0.2151
ETS	1.19	0.42	3.34	0.7441
BMI	0.94	0.83	1.05	0.2718
IgE total	1.00	1.00	1.00	0.2117
Cat*	0.90	0.32	2.53	0.8416
Dog*	3.25	0.32	32.68	0.3169
HDM*	0.39	0.15	1.00	**0.0497**
Molds*	0.39	0.09	1.65	0.2012
Grass*	0.43	0.17	1.10	0.0799
Tree*	1.03	0.36	2.96	0.9513
Food*	0.66	0.22	1.95	0.4475
Parental asthma	0.45	0.18	1.12	0.0876
Atopic dermatitis	0.64	0.22	1.88	0.4143
Allergic rhinitis	0.09	0.03	0.26	**0.0001**
Allergic rhinoconjuctivitis	0.05	0.01	0.38	**0.0043**
Animals at home	0.53	0.21	1.32	0.1729
Molds at home	1.00	0.13	7.47	1.0000

*API* Asthma Predictive Index; *BMI* Body mass index; *CI* Confidence interval; *ETS* Exposure to tobacco smoke; *HDM allergy* House dust mite allergy; *IgE* Immunoglobulin E; *OR* Odds ratio

^a^dependent variable: asthma remission vs. persistence asthma

^*^sensitization to allergens measured at baseline first visit

No association was found between asthma remission and lung functions parameters (Tables 3 and 4). Statistically significant correlation was found between asthma remission and FeNO but in lower values of FeNO only: Q2 vs. Q1 (OR: 4.77; 95% Cl: 1.14 to 19.98; *p* < 0.0327). The results are presented in Tables 3 and 4.

**Table 3:** Associations between asthma remission, defined as dependent variable and group of independent variables in univariate model of logistic regression analysis

		OR^a^	95% CI		*p*
FEV1 (% best)	Q2 vs. Q1	3.30	0.83	13.18	0.0911
	Q3 vs. Q1	1.54	0.37	6.45	0.5545
	Q4 vs. Q1	3.03	0.75	12.21	0.1200
FEV1/FVC (% best)	Q2 vs. Q1	1.25	0.31	5,07	0.7549
	Q3 vs. Q1	2.06	0.57	7.47	0.2698
	Q4 vs. Q1	1.04	0.28	3.92	0.9550
RTOT (% best)	Q2 vs. Q1	0.45	0.11	1.92	0.2837
	Q3 vs. Q1	1.13	0.29	4,41	0.8658
	Q4 vs. Q1	1.13	0.29	4.41	0.8658
ROCC (% best)	Q2 vs. Q1	2.86	0.72	11.31	0.1348
	Q3 vs. Q1	0.44	0.10	1.93	0.2762
	Q4 vs. Q1	1.79	0.47	6.82	0.3966
FeNO (ppb)	Q2 vs. Q1	4.77	1.14	19.98	**0.0327**
	Q3 vs. Q1	0.92	0.23	3.70	0.9028
	Q4 vs. Q1	1.47	0.38	5.72	0.5814
PPAR (%)	Q2 vs. Q1	1.00	0.28	3.54	1.0000
	Q3 vs. Q1	0.55	0.16	1.91	0.3440
	Q4 vs. Q1	0.36	0.10	1.29	0.1174
CD25 (%)	Q2 vs. Q1	1.36	0.39	4.79	0.6340
	Q3 vs. Q1	1.22	0.35	4.24	0.7516
	Q4 vs. Q1	0.91	0.26	3.20	0.8821
FOXP3 (%)	Q2 vs. Q1	1.22	0.35	4.24	0.7516
	Q3 vs. Q1	1.00	0.29	3.48	1.0000
	Q4 vs. Q1	1.83	0.52	6.43	0.3440
SOCS3 (%)	Q2 vs. Q1	1.10	0.31	3.88	0.8821
	Q3 vs. Q1	1.63	0.47	5.60	0.4380
	Q4 vs. Q1	1.22	0.35	4.24	0.7516
CD25CD71 (%)	Q2 vs. Q1	0.74	0.21	2.64	0.6431
	Q3 vs. Q1	0.98	0.27	3.52	0.9726
	Q4 vs. Q1	0.89	0.24	3.24	0.8584
GARP (%)	Q2 vs. Q1	0.60	0.17	2.11	0.4220
	Q3 vs. Q1	0.90	0.26	3.07	0.8665
	Q4 vs. Q1	0.82	0.24	2.84	0.7516

*CI* Confidence interval; *FeNO* Fractional exhaled nitric oxide; *FEV1* Forced expiratory volume in 1 s; *FOXP3* Forkhead transcription factor; *FVC* Forced vital capacity; *GARP* Glycoprotein A repetitions predominant; *OR* Odds ratio; *PPAR* Peroxisome proliferator-activated receptor; *Q1–Q4* Quartile 1–4; *ROCC* Receiver operating characteristic curve; *RTOT* Receivable turnover time ratio; *SOCS* Suppressor of cytokine signaling

^a^dependent variable: asthma remission vs. persistence asthma

**Table 4:** Comparisons of inflammatory parameters between presence and absence of clinical predictors of asthma remission

	FeNO (ppb)			PPAR (%)			CD25 (%)			FOXP3 (%)			SOCS3 (%)			CD25CD71 (%)			GARP (%)		
	Q25	M	Q75	Q25	M	Q75	Q25	M	Q75	Q25	M	Q75	Q25	M	Q75	Q25	M	Q75	Q25	M	Q75
API (allergy)																					
Absence	13.8	17.9	21.8	5.6	13.3	25.2	0.7	1.1	1.5	16.5	40.0	62.6	36.6	56.5	73.4	1.1	1.7	3.2	7.7	12.8	18.7
Presence	15.1	21.7	29.2	7.4	16.2	25.3	0.9	1.2	1.6	11.0	23.3	52.9	24.8	48.3	80.0	1.4	2.3	5.8	8.8	14.8	29.7
* p* level	0.1311			0.6742			0.3087			0.1734			0.5616			0.0755			0.3572		
* p* adjusted	0.4046			0.6742			0.500			0.4046			0.6552			0.4046			0.500		
API (eosinophils)																					
Absence	14.8	18.1	26.0	9.2	15.4	25.4	0.9	1.2	1.6	14.8	27.3	62.3	26.6	51.9	76.4	1.3	2.1	3.5	7.5	12.5	20.2
Presence	15.0	21.2	39.6	5.2	14.3	24.9	0.8	1.0	1.4	9.2	25.6	55.0	27.9	49.8	77.3	1.4	3.0	7.4	10.0	17.9	24.6
* p* level	0.3836			0.5929			**0.0476**			0.5585			0.992			0.2071			0.2607		
* p* adjusted	0.6713			0.6917			0.3332			0.6917			0.992			0.6083			0.6083		
API (wheez)																					
Absence	15.4	17.7	26.0	5.6	14.3	26.4	0.9	1.3	1.7	23.6	44.6	64.9	25.8	41.8	58.2	1.9	3.3	5.3	8.8	14.3	34.9
Presence	14.8	19.6	28.7	8.9	15.5	24.8	0.9	1.1	1.5	9.5	20.2	51.0	28.3	56.5	80.9	1.3	2.1	3.7	8.2	14.3	21.7
* p* level	0.851			0.6268			0.3133			**0.0158**			0.2074			0.1045			0.7345		
* p* adjusted	0.851			0.851			0.5482			0.1106			0.4839			0.3657			0.851		
HDM allergy																					
Absence	13.8	17.4	22.2	7.1	13.4	24.6	0.9	1.2	1.6	13.3	27.7	62.3	29.1	51.7	74.3	1.2	1.8	3.0	7.0	12.7	19.6
Presence	17.8	23.9	39.6	7.4	19.6	27.3	0.8	1.0	1.6	9.5	23.3	52.9	21.3	51.3	77.6	1.9	3.3	7.4	9.8	17.6	34.9
* p* level	**0.0061**			0.1918			0.3759			0.6514			0.96			**0.0232**			0.0591		
* p* adjusted	**0.0427**			0.3356			0.5262			0.7599			0.96			0.0812			0.1379		
AR																					
Absence	15.4	19.2	28.7	5.6	13.4	24.8	0.9	1.2	1.5	14.4	25.1	62.3	28.3	51.7	75.1	1.3	2.1	3.3	9 4	15.1	22.6
Presence	13.3	18.2	28.5	14.3	22.3	25.4	0.7	1.1	2.8	9.5	29.2	52.9	13.8	58.2	80.9	1.8	3.7	7.7	3.1	8.8	16.1
* p* level	0.5829			**0.0493**			0.9901			0.897			0.9263			**0.0198**			**0.0215**		
* p* adjusted	0.9901			0.1150			0.9901			0.9901			0.9901			0.0752			0.0752		

Data are presented as: *M* Median value, *Q25* Lower quartile, *Q75* Upper quartile and compared with Mann–Whitney test. *p*-adjusted according to Benjamini and Hochberg correction for multiple comparisons

*AR* Allergic rhinitis; *FeNO* Fractional exhaled nitric oxide; *FOXP3* Forkhead transcription factor; *GARP* Glycoprotein A repetitions predominant; *HDM* House dust mite allergy; *PPAR* Peroxisome proliferator-activated receptor; *ppb* Part per billion; *SOCS* Suppressor of cytokine signaling

In the second step, inflammatory/immunoregulatory data (categorized according to lower and upper quartiles) as independent variables were included in the univariate model of logistic regression analysis together with asthma remission as dependent variable (Supplementary Table S1). Only in lower values, the FeNO level was associated with asthma remission as described above (Supplementary Table S1). The second versus the first quartile of FeNO increased the prevalence of asthma remission, but the third and fourth did not affect it (Supplementary Table S1). No correlation was found between immunological parameters and asthma remission (Supplementary Table S1).

Finally, all statistical predictors were included in the multivariate logistic regression analysis. Only the current allergic rhinitis independently decreased the probability of asthma remission (OR: 0.15; 95% CI: 0.039 to 0.56).

The presence of increased eosinophil blood count in Asthma Predictive Index (API) criteria was associated with a lower expression of CD25 positive cells but it was not present after correction for multiple comparisons (Table 4). Current allergy to HDM was associated with a higher FeNO level (before and after correction for multiple comparisons), and a higher expression of CD25CD71 positive cells (only before correction for multiple comparisons). Allergic rhinitis was associated with a higher expression of PPAR and CD25CD71 positive cells; in the same group of patients, a lower expression of GARP positive cells were observed (all associations were present only before correction for multiple comparisons).

**Fig. 1:**
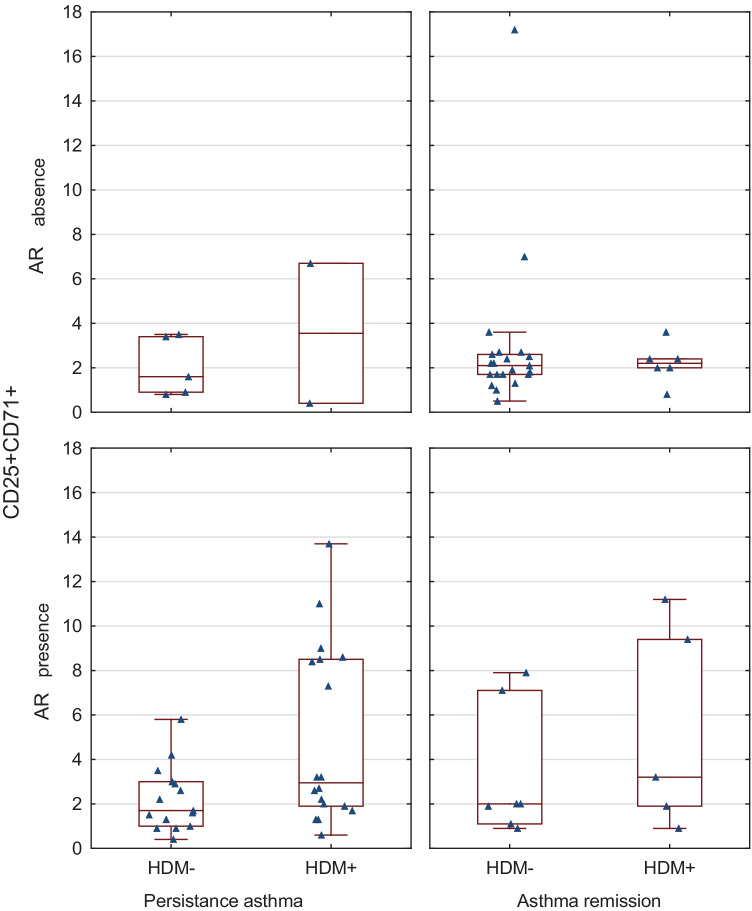
Allergy to house dust mite allergens (HDM) and the presence of allergic rhinitis (AR) symptoms determine the expression of CD25 + CD71 + cells in patients with/without asthma

Allergy to HDM and the presence of allergic rhinitis symptoms determine the expression of CD25CD71 cells in patients with/without asthma (Fig. 1). A higher expression of CD25CD71 in peripheral blood mononuclear cells was seen in patients with AR with hypersensitivity to HDM. The persistence of childhood asthma was largely determined by the presence of allergic rhinitis and sensitization to HDM.

## Discussion

Little is known about the immune mechanisms by which asthma progresses to persistent or regression of symptoms [18, 19]. A part of children with asthma will “outgrow” the disease, and become symptom-free as adults, although many remain overactive [20–22]. The present study was done to better understand the mechanism why some preschoolers develop persistent asthma in school age, while others outgrow it.

In the present study, allergic rhinitis was a strong predictor of asthma persistence into adulthood. Hypersensitivity to HDM, higher level of exhaled NO (weak association) as well as mAPI criteria (such as sensitization to aeroallergens, blood eosinophils, wheezing apart from colds) were associated with persistent disease.

The expression of all immunological parameters between both groups (asthma remission vs. asthma persistence) was comparable which suggests that natural remission of clinical symptoms of asthma in children is not related to immunoregulatory processes, and only follows the clinical and allergic status. However, immunoregulatory parameters such as CD25, CD25CD71, PPAR, GARP, and FOXP3 positive cells are associated with the occurrence of allergic rhinitis, hypersensitivity to mites, as well as increased eosinophil counts and wheezing in addition to colds in API, suggesting their possible role in the regulation of peripheral tolerance. In the present study, the combination of rhinitis, and HDM allergy in particular, predicted the persistence of asthma in school-age children. Many studies have looked at the prognostic factors of remission or persistence of asthma from early childhood to adulthood [23–27]. Observations in this study, especially those regarding immunology parameters, were surprising: presence of allergic rhinitis correlated with PPAR, CD25CD71, and GARP. Most of the glycoprotein A (GARP) repeats have a strong anti-inflammatory and regulatory effect on human cells in vitro and in vivo and it is involved in the regulation of peripheral [10]. The authors showed that sGARP acted synergistically with Treg to prevent inflammation [10]. In the present study higher expression of GARP positive cells were shown in patients without AR. Khare et al. showed that CD11c-specific PPARγ deficiency disturbs de novo FOXP3 expression in CD4 + T cells and enhances the expression of proinflammatory cytokines in CD11c + cells, thus disrupting the induction of airway tolerance [28]. A recent study by Chen et al. has showed that PPAR-γ also acts as a driving factor for type 2 responses in allergy [7]. The increased expression of CD71 may reflect the increased activation state [6]. In the present study, a higher expression of PPARγ and CD25CD71 was demonstrated in peripheral blood mononuclear cells of patients with allergic rhinitis compared to children without AR.

Additionally, a higher expression of CD25CD71 was found in children with hypersensitivity to HDM. The study has shown an association between asthma remission, HDM allergy, the presence of allergic rhinitis and the expression of CD25CD71 (Fig. 1). It is clearly visible that HDM allergy and the presence of allergic rhinitis, rather than asthma remission, determine CD25CD71 expression. FOXP3 expression in Tregs plays a key role in maintaining immune tolerance.

In the present study, API parameters correlated with higher level of CD25 positive regulatory T cells. Suppressor of silencing cytokine signaling 3 (SOCS3) weakens the functions of eosinophils, the key inflammatory cells in asthma, but in the present study, it did not correlate with the studied clinical factors.

The present study has some limitations. While childhood asthma was considered to be a heterogeneous phenotype, the study was unable to differentiate asthma by phenotype only to characterize factors that contribute to the persistence or remission of asthma. In addition, children with and without allergic rhinitis were allowed to take nasal steroids that may be sufficient to treat particularly mild asthma, were classified as remission group. Levels of immunological markers change with patient’s age, and only few have standard ranges. All immunological measurements were performed when the children were older; therefore, all findings are related to the present clinical outcomes. With much larger group of children, the trend of changes and some correlations of immunological markers and asthma remission/persistence could probably be found.

## Conclusion

The likelihood of persistence of childhood is determined by the presence of allergic rhinitis and sensitization to HDM. Significant negative correlation between asthma remission and API criteria such as wheezing apart from colds, elevated blood eosinophils count, and sensitization to aeroallergens was observed. The present results suggest that natural remission of clinical symptoms for asthma in children may not be fully related to immunoregulation processes. Future research on asthma should be longitudinal and integrate various approaches for both pediatric and adult populations [29].

## Supplementary Information

Below is the link to the electronic supplementary material.

Supplementary file1 (DOCX 16 KB)
