# Supplementary material for: Allergic Rhinitis and House Dust Mite Sensitization Determine Persistence of Asthma in Children
Source: Indian J Pediatr. 2022 Mar 4;89(7):673–81. doi: 10.1007/s12098-021-04052-5 (PMC9205813; doi:10.1007/s12098-021-04052-5)
Supplement: Supplementary file 1 — Supplementary file1 (DOCX 16 KB) [file 12098_2021_4052_MOESM1_ESM.docx]

**Supplementary material S1** Immunological assessment

The following panel of antibodies conjugated with fluoresce in isothiocyanate (FITC), phycoerythrin (PE), Peridinin-chloro-phyll-protein (Per-CP), orallophycocyanin (APC) was used for assays: PPARG-FITC, CD11c-PE, CD 25 FITC, CD 4 PerCP, CD 71 PE, CD 73, PerCP, Anti- GARP APC, FOXP3 PE, (all antibodies from Becton Dickinson, San Diego, CA, USA) and SOCS3 (from Abbexa, Cambridge, UK). All procedures were carried out according to the manufacturer's instructions. The cells were incubated with monoclonal antibodies SOCS3, FOXP3, in the dark at room temperature for 30 minutes. Afterincubation, the cells were washed in PBS.

Cells for expression of SOCS3 were incubated with the FITC Swine Anti Rabbit 2nd order antibody at a 1:20 dilution (DAKO, Denmark), after incubation the cells were washed in PBS and assessed by flow cytometry (BD FACS Canto II-, San Diego, CA, USA).

The isolated mononuclear cells were incubated with surface monoclonal antibodies against CD25, CD4, CD71, CD73, GARP, PPARG, CD11c or intracellular SOCS3, FOXP3 in the dark, at room temperature for 30 minutes. The samples were then washed twice in buffered saline (PBS) (5 min 140 g) (PBS) (PAA Laboratories GmbH, Austria). Cells for evaluating the expression of intracellular antigens were fixed using an intracellular staining kit according to the manufacturer's protocol (BD, San Diego, CA, USA).

Tregs were assessed using the anti-CD25 (FITC-conjugated; Becton Dickinson, San Jose, CA, USA) anti-CD4 (PerCP-conjugated; Becton Dickinson, San Jose, CA, USA) and anti-human FOXP3 antibody monoclonalantibodies (PE-conjugated; Becton Dickinson, San Jose, CA, USA). The peripheral blood mononuclear cells (PBMCs) were isolated from whole blood by density gradient centrifugation on Pancoll human density-1077 (PAN Biotech, Germany) and cultured in standard mammalian cell culture conditions (37°C, 5% carbondioxide, 80% relative humidity) for 72 h, 2x106 cells/ml in the presence of allergen in concentration 750 SBU/mL. After cell culture cells were centrifuged and washed in the BD Pharmingen™ StainBuffer (FBS) (5 min/250 g). Then, they were incubated with 20 µl of anti-CD25/anti-CD4 monoclonal antibody cocktail (for 20 min, at room temperature, in the dark). Afterward, cells were washed in the BD Pharmingen™ Stain Buffer (FBS) and centrifuged (5 min/250 g). Cells were then re-suspended and incubated for 10 min with Human FoxP3 Buffer A (at room temperature, in the dark). After washing the cells twice the cells and discarding the supernatant, the sample was re-suspended in FoxP3 Buffer C. Incubated for 30 minutes at RT protected from light. After washing the cells twice the cells and discarding the supernatant, the sample was re-suspended in 20 µl of PE anti-human FOXP3 or the isotype control antibody was added and incubated for 30 min (at room temperature, in the dark). After that, cells were washed twice, re-suspended with cell staining buffer, and the fluorescence was measured using flow cytometry (FACS CantoII, BD Biosciences, SanJose, California). Cells expressing CD4+/CD25high+/FOXP3+ were determined as Tregs. The percentage of Tregs among mononuclear cells, which were gated based on SSC and FSC distribution, was calculated.

**Supplementary Table S1** Pulmonary function tests and laboratory results by study groups

| **Asthma persistence Asthma remission  *p*** | | | | | | | | | |
| --- | --- | --- | --- | --- | --- | --- | --- | --- | --- |
|  | ***N*** | **Median** | **Q25** | **Q75** | ***N*** | **Median** | **Q25** | **Q75** | ***p*** |
| **FEV1 (% pred)** | 37 | 101 | 90 | 114 | 35 | 106 | 100 | 118 | 0.1100 |
| **FEV1/FVC (% pred.)** | 38 | 102 | 95 | 109 | 35 | 102 | 96 | 108 | 0.9344 |
| **Rtot (% pred.)** | 35 | 185 | 152 | 225 | 31 | 202 | 151 | 228 | 0.3641 |
| **Rocc (% pred.)** | 37 | 167 | 140 | 195 | 33 | 159 | 146 | 203 | 0.7701 |
| **FeNO (ppb)** | 38 | 22 | 13 | 29 | 33 | 18 | 15 | 26 | 0.9043 |
| **PPAR (%)** | 40 | 21.2 | 11.3 | 25.8 | 40 | 13.2 | 5.2 | 20.4 | 0.0584 |
| **CD25 (%)** | 40 | 1.2 | 0.9 | 1.6 | 40 | 1.2 | 0.9 | 1.5 | 0.9580 |
| **FOXP3 (%)** | 40 | 25.6 | 10.8 | 53.1 | 40 | 27.3 | 15.9 | 62.6 | 0.6842 |
| **SOCS3 (%)** | 40 | 49.9 | 24.6 | 76.9 | 40 | 51.9 | 32.0 | 76.4 | 0.6983 |
| **CD25CD71 (%)** | 40 | 2.2 | 1.3 | 4.7 | 40 | 2.2 | 1.4 | 4.6 | 0.7704 |
| **GARP (%)** | 40 | 14.2 | 9.0 | 23.2 | 40 | 14.6 | 7.8 | 22.2 | 0.7558 |

*CD25* A type of  transmembrane protein present on activated T cells; *CD25CD71* Ratio of transmembrane protein present on activated T cells CD 25 and CD 71; *FeNO* Fractional exhaled nitric oxide; *FEV1* Forced expiratory volume in 1st second; *FEV1/FVC* Ratio of  forced expiratory volume in 1st second to Forced vital capacity; *FOXP3* Forkhead transcription  factor; *GARP* Glycoprotein A repetitions predominant; *PPAR*  Peroxisome proliferator-activated receptor gamma; *ROCC* Receiver operating characteristic  curve; *RTOT*  Receivable turnover time ratio; *SOCS3* Suppressor of cytokine signalling 3

Data are presented by median value with lower (Q25) and upper (Q75) quartiles
